# Supplementary material for: The genome sequence of the barkfly, Loensia variegata (Latreille, 1799) (Psocodea: Psocidae)
Source: Wellcome Open Res. 2026 Apr 16;11:224. [Version 1] doi: 10.12688/wellcomeopenres.26324.1 (PMC13219991; doi:10.12688/wellcomeopenres.26324.1)
Supplement: Peer review history

# Reviewer report: Reviewer response for version 1

**DOI:** 10.21956/wellcomeopenres.28991.r155294

**Published:** 2026-05-28

**Authors:** Markus Friedrich

**Affiliations:** 1 Wayne State University, Detroit, Michigan, USA

This submission reports the sequencing and assembly of the nuclear and mitochondrial genome sequences of the barkfly, Loensia variegata, using a single adult male as sample input.

 1. “DNA was sheared into an average fragment size of 12–20 kb We used…”

 Add period after “kb”

 2. “The iuLoeVari1 sample was weighed and triaged to determine the appropriate extraction protocol.”

 &

 “The Hi-C sample was prepared from 20–50 mg of frozen tissue from the iuLoeVari1 sample using the Arima-HiC v2 kit (Arima Genomics).”

 20–50 mg strikes me as high for a single barklfy specimen.

 What was the initial weight of the sample determined before optimizing the extraction protocol?

 3. “The X chromosome was assigned based ony read coverage,...”

 ony = on?

 4. “Closely related species are known to have the XO karyotype.”

 Add reference or species genome assemblies.

 5. “The mitochondrial genome was also assembled (length 15.94 kb, OZ180003.1). This sequence is included as a contig in the multifasta file of the genome submission and as a standalone record.”

 Add sequence ID of the standalone mt genome sequence submission.

Are sufficient details of methods and materials provided to allow replication by others?

Yes

Is the rationale for creating the dataset(s) clearly described?

Yes

Are the datasets clearly presented in a useable and accessible format?

Yes

Are the protocols appropriate and is the work technically sound?

Yes

Reviewer Expertise:

Comparative genomics and gene family evolution.

I confirm that I have read this submission and believe that I have an appropriate level of expertise to confirm that it is of an acceptable scientific standard.

# Reviewer report: Reviewer response for version 1

**DOI:** 10.21956/wellcomeopenres.28991.r155293

**Published:** 2026-05-25

**Authors:** Mireille Steck

**Affiliations:** 1 University of Hawaiʻi at Mānoa, Honolulu, USA

This is a fine addition for a genomic assembly of a barkfly following appropriate genomic assembly methods. Clear cutoffs and assembly statistics have been well reported. The genome assembly looks to be complete and well annotated. This genome will provide important metrics for future insect evolution studies and is a great contribution to the field.

Are sufficient details of methods and materials provided to allow replication by others?

Yes

Is the rationale for creating the dataset(s) clearly described?

Yes

Are the datasets clearly presented in a useable and accessible format?

Yes

Are the protocols appropriate and is the work technically sound?

Yes

Reviewer Expertise:

Molecular Biology

I confirm that I have read this submission and believe that I have an appropriate level of expertise to confirm that it is of an acceptable scientific standard.
